# Parallel Evolution to Elucidate the Contributions of PA0625 and *parE* to Ciprofloxacin Sensitivity in *Pseudomonas aeruginosa*

**DOI:** 10.3390/microorganisms11010013

**Published:** 2022-12-21

**Authors:** Qi Liu, Liwen Yin, Chenjing Lv, Fang Bai, Zhihui Cheng, Weihui Wu, Yongxin Jin

**Affiliations:** State Key Laboratory of Medicinal Chemical Biology, Key Laboratory of Molecular Microbiology and Technology of the Ministry of Education, Department of Microbiology, College of Life Sciences, Nankai University, Tianjin 300071, China

**Keywords:** *Pseudomonas aeruginosa*, ciprofloxacin resistance, PA0625, pyocin, *ParE*

## Abstract

*Pseudomonas aeruginosa* is a ubiquitous pathogen that causes a wide range of acute and chronic infections. Ciprofloxacin, one of the first-line fluoroquinolone class antibiotics, is commonly used for the treatment of *P. aeruginosa* infections. However, ciprofloxacin-resistant *P. aeruginosa* is increasingly reported worldwide, making treatment difficult. To determine resistance-related mutations, we conducted an experimental evolution using a previously identified ciprofloxacin-resistant *P. aeruginosa* clinical isolate, CRP42. The evolved mutants could tolerate a 512-fold higher concentration of ciprofloxacin than CRP42. Genomic DNA reference mapping was performed, which revealed mutations in genes known to be associated with ciprofloxacin resistance as well as in those not previously linked to ciprofloxacin resistance, including the *ParE*^R586W^ substitution and PA0625 frameshift insertion. Simulation of the *ParE*^R586W^ substitution and PA0625 frameshift insertion by gene editing in CRP42 and the model strain PAO1 demonstrated that while the PA0625 mutation does contribute to resistance, mutation in the *ParE*^R586W^ does not contribute to resistance but rather affects tolerance against ciprofloxacin. These findings advance our understanding of ciprofloxacin resistance in *P. aeruginosa*.

## 1. Introduction

*Pseudomonas aeruginosa* is a Gram-negative opportunistic human pathogen with high prevalence in hospitals. It is a common cause of acute and chronic infections in individuals with cystic fibrosis (CF) or hospitalized in intensive care units [1]. It is recognized as the critical priority tier of antibiotic-resistant pathogens by the World Health Organization for the research and development of new antibiotics [2]. It is also included in the group of “ESKAPE” pathogens, which comprise *Enterococcus faecium*, *Staphylococcus aureus*, *Klebsiella pneumoniae*, *Acinetobacter baumannii*, *P. aeruginosa*, and *Enterobacter* species, for which novel therapeutic options are urgently needed [3].

Ciprofloxacin is one of the first-line fluoroquinolone class antibiotics used to treat a wide range of infections by *P. aeruginosa* [4]. However, an increasing proportion of clinical *P. aeruginosa* isolates have been found to be resistant to ciprofloxacin [5]. Multiple mechanisms of ciprofloxacin resistance have been demonstrated in *P. aeruginosa*, mainly including (i) mutations in the ciprofloxacin target-encoding genes *gyrA*/*gyrB* (encoding DNA gyrase) or *parC*/*parE* (encoding topoisomerase IV) to reduce the affinity of targets for ciprofloxacin [6,7]; (ii) mutations in the regulatory genes resulting in overexpression of efflux pumps to increase the expulsion of ciprofloxacin, including *mexS* (repressor of *mexEF-oprN*), *nfxB* (repressor of *mexCD-oprJ*), *mexZ* (repressor of *mexXY*), and *mexR* (repressor of *mexAB-oprM*) [4,8]; and (iii) acquisition of ciprofloxacin modifying genes through plasmid-mediated gene transfer [9]. Novel mutations and genes associated with ciprofloxacin resistance were discovered using a laboratory experimental evolution approach to generate ciprofloxacin-resistant mutants from sensitive strains of *P. aeruginosa* in combination with whole-genome sequencing [10,11]. Although how these mutations contribute to resistance against ciprofloxacin in *P. aeruginosa* has not been determined, these studies reveal the high potential of parallel evolution to uncover previously unknown resistance genes.

In this study, we employed experimental evolution and genomic DNA reference mapping to identify resistance genes for ciprofloxacin in *P. aeruginosa*. We found mutations in genes known to be associated with ciprofloxacin resistance. Our results also revealed mutations in two genes not previously linked to ciprofloxacin resistance, including the *ParE*^R586W^ substitution and PA0625 frameshift insertion.

## 2. Materials and Methods

### 2.1. Bacterial Strains, Plasmids, Primers, and Culture Conditions

The bacterial strains, plasmids, and primers used in the present study are shown in Tables S1 and S2. The *P. aeruginosa* strain CRP42 was isolated from the sputum sample of a patient [8]. Bacterial cells were cultured in Luria-Bertani (LB) broth (5 g/L yeast extract, 5 g/L NaCl, and 10 g/L tryptone) or on LB agar plates (supplemented with 15 g/L agar) at 37 °C. When needed, concentrations of tetracycline were used at 10 µg/mL in *E. coli* and 50 µg/mL in *P. aeruginosa*. The minimum inhibitory concentration (MIC) of ciprofloxacin was determined by a 2-fold serial dilution method with modifications [12]. Briefly, each well of a 96-well microtiter plate was filled with 100 µL LB broth with serially diluted concentrations of ciprofloxacin. One hundred microliters of bacterial culture (10^4^ CFU) were inoculated into each well. Growth was assessed visually after 20 h of incubation at 37 °C. All strains were tested in triplicate.

### 2.2. Parallel Evolution to Select for Ciprofloxacin-Resistant Mutants

The continuous ciprofloxacin evolution experiment with three repeats was carried out in LB broth containing increasing concentrations of ciprofloxacin. On day 1, overnight cultures of CRP42 were inoculated (100-fold dilution) into four tubes containing 2, 4, 8, and 16 µg/mL ciprofloxacin, which correspond to 0.25×, 0.5×, 1×, and 2× MIC for CRP42, respectively. After 24 h of aerobic culture at 37 °C, cells from each population with the highest concentration of ciprofloxacin that allowed bacterial growth to an optical density at 600 nm above 1 were measured for the MIC and reinoculated into fresh medium as before, containing 0.25×, 0.5×, 1×, and 2× MIC (obtained one day earlier). This serial passaging was repeated until the MIC of the evolved strains rose to 4096 µg/mL. Another three repeats were passaged in LB broth medium serving as controls.

### 2.3. Plasmid Construction and Gene Editing

To make the *parE* (*ParE* R586W) point mutation construct, a 1553 bp DNA fragment with the *parE*^R586W^ site located in the middle region was PCR amplified using R3 genomic DNA as a template (primers in Table S2), digested with *Sac*I-*Hin*dIII, and then ligated into the pEX18Tc suicide vector [13], leading to pEX18-*parE*^R586W^. Gene replacement in CRP42 or PAO1 was conducted by conjugally transferring pEX18-*parE*^R586W^ followed by selection for single crossover and then double crossover [13]. The resulting strain CRP42*parE*^R586W^ or PAO1*parE*^R586W^ was confirmed by PCR amplification and sequencing analysis (primers in Table S2). Plasmids pEX18-*nfxB*^G180S^, pEX18-*nfxB*^X188C^, and pEX18-PA0625_R3_ were generated with similar manipulations as pEX18-*parE*^R586W^. CRP42*nfxB*^G180S^, CRP42*nfxB*^X188C^, CSP18*mexS*_CRP42_*nfxB*^G180S^, CSP18*mexS*_CRP42_*nfxB*^X188C^, CRP42PA0625_R3_, and PAO1PA0625_R3_ were constructed with similar manipulations as CRP42*parE*^R586W^.

To delete the PA0625 gene, a 762 bp fragment immediately upstream of the PA0625 gene and a 670 bp fragment downstream of the PA0625 gene were amplified using PAO1 or CRP42 genomic DNA as templates (primers in Table S2). These two fragments were digested with *Eco*RI-*Bam*HI and *Bam*HI-*Hin*dIII and then ligated into pEX18Tc, resulting in pEX18-PA0625-1 (PAO1 genomic DNA as template) and pEX18-PA0625-2 (CRP42 genomic DNA as template). Gene deletion in PAO1 or CRP42 was performed with pEX18-PA0625-1 or pEX18-PA0625-2 as described above.

### 2.4. Genomic DNA Isolation and Reference Mapping

Genomic DNAs of R1, R2, and R3, as well as control strains C1, C2, and C3, were extracted from overnight cultures of bacterial cells using a DNA purification kit (Tiangen Biotec, Beijing, China). Genomic DNA reference mapping was carried out by GENEWIZ Life Sciences (Suzhou, China) as described in our previous studies [8,14]. The raw sequence data have been deposited in the NCBI database with accession number PRJNA875020.

### 2.5. Ciprofloxacin Susceptibility Assay

Overnight cultures of *P. aeruginosa* strains were diluted 50-fold into LB broth medium and grown at 37 ℃ to an OD_600_ of 1.0. The bacterial cells were treated with ciprofloxacin at a final concentration of 16 μg/mL (for CRP42 and CRP42*parE*^R586W^) or 0.25 μg/mL (for PAO1 and PAO1*parE*^R586W^) and then further cultured at 37 ℃ with agitation (200 rpm) for up to 6 h. The number of viable bacterial cells was determined by serial dilution and plating at the indicated time. The bacterial survival% = (live bacterial number after ciprofloxacin treatment) × 100/(initial inoculum bacterial number).

### 2.6. Statistical Analysis

GraphPad Prism 7.0 software was used to conduct the statistical analyses. *P* values were calculated using the two-tailed unpaired Student’s *t* test. Differences were considered statistically significant when the *P* value was below 0.05.

## 3. Results

### 3.1. Development of Resistance to Ciprofloxacin by the Clinical Isolate CRP42

To uncover novel ciprofloxacin resistance-related genes and understand the evolutionary characteristics of clinically resistant *P. aeruginosa*, we utilized strain CRP42, a ciprofloxacin-resistant clinical isolate due to the *GyrA* D87G mutation and overexpression of the MexEF-OprN efflux pump [8], to perform parallel evolution. The development of ciprofloxacin resistance was carried out by the daily passage of three biologically replicate populations of CRP42 in LB broth medium containing increasing concentrations of ciprofloxacin. After 17–34 days of evolution, the MICs for the three groups reached 4096 µg/mL (a 512-fold increase compared to CRP42 in Figure S1), whereas no change in MIC was detected for the three control groups that were evolved in the antibiotic-free LB broth for the same period. To validate that the resistance is caused by stable mutations, we streaked the population of each replicate that reached the highest MICs at the evolutionary end on LB agar plates. A random colony was isolated from each of the plates and designated R1, R2, and R3. The strains were cultured in LB broth medium for two sequential passages, and the MICs were determined with the 2-fold dilution method. The MICs of these strains were the same as those of the corresponding populations (Table 1), suggesting that increased resistance to ciprofloxacin was due to stable mutations.

### 3.2. Candidate Mutations Related to Ciprofloxacin Resistance in the Evolved Strains

To determine the genetic events that contribute to the increased ciprofloxacin resistance in the evolved mutants, we conducted genomic DNA reference mapping for the R1, R2, and R3 strains, as well as the control strains C1, C2, and C3. Table 2 shows the candidate mutations related to ciprofloxacin resistance in the evolved strains that were not present in the C1, C2, or C3 strains (all mutated genes are listed in Table S3). The threonine at position 83 of *GyrA* was substituted with isoleucine, alanine, and valine in the R1, R2, and R3 strains, respectively (Table 2). It has been reported that T83 plays a key role in the binding of ciprofloxacin by *GyrA* of *P. aeruginosa* [4]. *GyrA* variants with T83I and T83A substitutions have been associated with ciprofloxacin resistance in *P. aeruginosa* clinical isolates and in vitro-evolved resistant mutants [15,16]. In addition to *GyrA*, mutations were also observed in *GyrB* (a proline insertion after codon A458 in R2), *ParC* (substitutions E91K in R1 and S87L in R3), and *ParE* (EV insertion before codon D450 in R1, VD insertion before codon G451 in R2, and R586W substitution in R3). All three strains also carried mutations in the *nfxB* gene (stop codon changed to cysteine in R1 and R2, and G180S substitution in R3), which encodes a repressor for the efflux pump MexCD-OprJ [17]. G180S substitution and stop codon loss mutation (stop codon changed to arginine) have been reported to impair *NfxB* repressor activity, leading to ciprofloxacin resistance in the strain PAO1 [18]. In line with a previous study [18], our simulated replacements of *nfxB* increased the MIC of ciprofloxacin in both CRP42 and CSP18*mexS*_CRP42_, a strain with MexEF-OprN efflux pump overproduction [8] (Table 1). In addition, strain R3 carried a frameshift insertion in PA0625, which encodes a possible lytic system of R-type pyocin [19,20].

### 3.3. The ParE^R586W^ Substitution Is Not Responsible for Resistance, but Tolerance to Ciprofloxacin in P. aeruginosa

Mutations in the genes encoding *GryA*/*B* and *ParC*/*E* are major contributors to ciprofloxacin resistance in *P. aeruginosa* [4]. The alterations in the quinolone resistance determining region (QRDR) in *GyrA*/*B* and *ParC*/*E* caused by mutations have been reported to contribute to ciprofloxacin resistance in *P. aeruginosa* [6,21]. All the mutations in *GyrA*/*B* and *ParC*/*E* in the R1, R2, and R3 strains, except for the *ParE*^R586W^ mutation in R3, are located in the QRDR [21]. Therefore, we wanted to investigate whether the R586W substitution in *ParE* plays a role in ciprofloxacin resistance. The chromosomal *parE* gene of the parental strain CRP42 was replaced by *parE*_R3_, and the MIC of ciprofloxacin was measured. As shown in Table 1, the replacement of *parE* with *parE*_R3_ had no impact on the MIC of CRP42. The R586W mutation was further generated in *ParE* of the model strain PAO1. Consistent with the unchanged MIC of CRP42*parE*^R586W^, the PAO1*parE*^R586W^ strain displayed the same MIC against ciprofloxacin as its parental strain PAO1 (Table 1). These results suggested that the *ParE*^R586W^ substitution is not responsible for ciprofloxacin resistance in *P. aeruginosa.*

**Figure 1:**
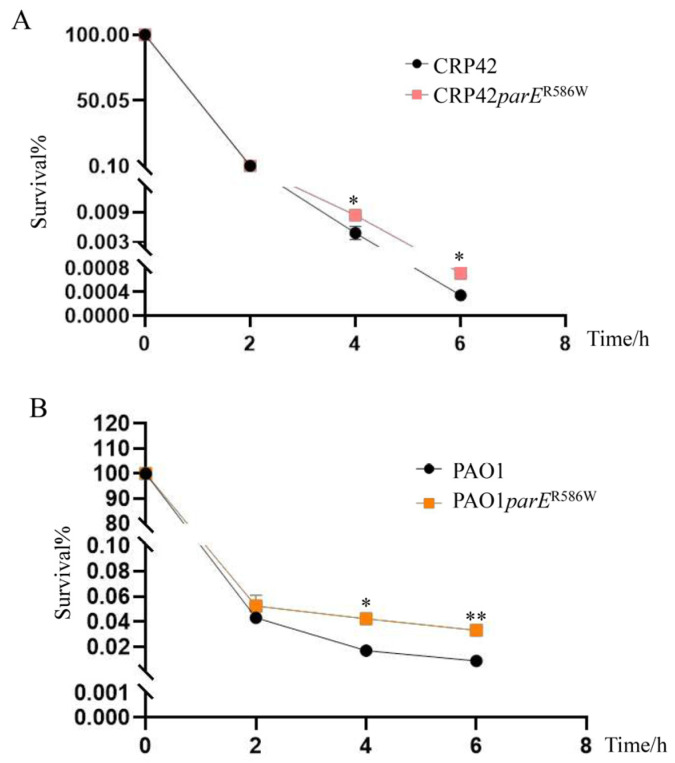
Roles of the *ParE*^R586W^ mutation in the bacterial tolerance to ciprofloxacin. Bacterial cultures at an OD_600_ of 1.0 were treated with (**A**) 16 µg/mL and (**B**) 0.25 µg/mL ciprofloxacin in LB broth with agitation. Bacterial cells were collected at the indicated time points, and the survival% were determined by serial dilution and plating. The data represent the results from three independent experiments, and the error bars represent standard deviations. *, *P* < 0.05, **; *P* < 0.01, by Student’s *t* test.

Next, we examined the role of the *ParE*^R586W^ mutation in the tolerance of *P. aeruginosa* against ciprofloxacin using the killing assay. Ciprofloxacin concentrations at 2 × MICs for CRP42 and CRP42*parE*^R586W^ (16 μg/mL) or PAO1 and PAO1*parE*^R586W^ (0.25 μg/mL) were used. As shown in Figure 1, the *ParE*^R586W^ substitution significantly increased the bacterial survival rate in both the PAO1 and CRP42 strains.

### 3.4. PA0625 Mutation Contributes to Ciprofloxacin Resistance in P. aeruginosa

The region from PA0613 to PA0648 encodes bacteriophage-like pyocins [22]. Expression of the genes in this region, including PA0625, was induced by ciprofloxacin [22]. Mutations in some genes in this region have been found to increase resistance to ciprofloxacin in both PAO1 and PA14 strains [22,23]. Our reference mapping and sequencing analysis for the PA0625 amplicon of the R3 strain revealed that R3 carries a frameshift insertion in PA0625. However, the contribution of PA0625 to ciprofloxacin resistance has not been established in *P. aeruginosa*. To test whether the PA0625 mutation increased the resistance to ciprofloxacin in *P. aeruginosa*, we replaced the PA0625 gene with PA0625_R3_ and deleted PA0625 in both the CRP42 and PAO1 strains using gene recombination. As shown in Table 1, the deletion or replacement of PA0625 with PA0625_R3_ resulted in a 1.2-fold and a 2-fold increase in the MIC of ciprofloxacin in CRP42 and PAO1, respectively. These results demonstrated that PA0625 is involved in ciprofloxacin resistance in *P. aeruginosa*.

## 4. Discussion

*P. aeruginosa* can cause serious nosocomial infections, and treatment is challenging. Ciprofloxacin, one of the first-line fluoroquinolone class antibiotics, has become an important tool in treating *P. aeruginosa* infections, but increasing resistance threatens its efficacy. Demonstrating novel resistance-related mutations during the development of bacterial resistance to ciprofloxacin may provide clues for novel strategies to suppress resistance evolution. In this study, we combined an in vitro evolution assay with genomic DNA reference mapping to determine resistance-related mutations. We found that mutations occur in genes known to be associated with ciprofloxacin resistance. Our results also revealed that the PA0625 mutation contributes to ciprofloxacin resistance in *P. aeruginosa*, while the *ParE*^R586W^ substitution confers *P. aeruginosa* tolerance to ciprofloxacin.

*parE* encodes the *ParE* subunit of topoisomerase IV in *P. aeruginosa*. *ParE* variants with amino acid changes of D419N, E459D, A473V, and S457R have been shown to be associated with fluoroquinolone resistance in *P. aeruginosa* [4,6,7]. Mutations in the *parE* gene for fluoroquinolone resistance are rare compared to those in *gyrA* and *parC*, perhaps because alterations in the sequence of *ParE* confer lower-level resistance against fluoroquinolone [4,7]. In this study, we demonstrated that the *ParE* R586W substitution, which was located away from the QRDR, does not contribute to resistance but confers tolerance against ciprofloxacin in *P. aeruginosa*. Since antibiotic tolerance mutations facilitate the rapid evolution of resistance in *E. coli* [24], it is possible that the *ParE*^R586W^ mutation paves the way for the subsequent evolution of ciprofloxacin resistance in *P. aeruginosa*.

Pyocins are bacteriocins that are synthesized by more than 90% of *P. aeruginosa* strains [19]. R-type pyocins are able to kill other Gram-negative bacteria in a bacterial niche [19]. Production and release of pyocins lead to lysis of the producer cells [20]. Pyocin biosynthesis genes contribute to bacterial susceptibility to ciprofloxacin because mutations in some of those genes increase resistance to ciprofloxacin and other fluoroquinolones in *P. aeruginosa* [22]. Our previous studies showed that upregulation of pyocin biosynthesis genes contributes to increased susceptibility to ciprofloxacin [25,26], while downregulation of pyocin biosynthesis genes increases ciprofloxacin resistance in *P. aeruginosa* [27]. Here, we showed that the PA0625 mutation conferred increased ciprofloxacin resistance in *P. aeruginosa.* Given the importance of cell lysis in R-type pyocin-mediated susceptibility to ciprofloxacin and the fact that PA0625 likely encodes a lytic system similar to those of bacteriophages [20,22], it is possible that a mutation in PA0625 disabled bacterial cell lysis by R-type pyocin in the presence of ciprofloxacin in *P. aeruginosa*.

In summary, we discovered two novel mutations that contribute to *P. aeruginosa* resistance to ciprofloxacin, including PA0625, which directly contributes to resistance, and the *ParE* R586W substitution, which contributes to tolerance against ciprofloxacin. These findings advance our understanding of ciprofloxacin resistance in *P. aeruginosa*.

## Figures and Tables

**Table 1:** MICs (μg/mL) of indicated *P. aeruginosa* strains.

Strains	Ciprofloxacin (μg/mL)
CRP42	8
R1	4096
R2	4096
R3	4096
CRP42*nfxB*^G180S^	16
CRP42*nfxB*^X188C^	16
CSP18*mexS*_CRP42_	2
CSP18*mexS*_CRP42_*nfxB*^G180S^	4
CSP18*mexS*_CRP42_*nfxB*^X188C^	4
PAO1	1/8
PAO1*parE*^R586W^	1/8
PAO1PA0625_R3_	1/4
PAO1ΔPA0625	1/4
CRP42*parE*^R586W^	8
CRP42PA0625_R3_	9.5
CRP42ΔPA0625	9.5

**Table 2:** Candidate mutations related to ciprofloxacin resistance in experimentally evolved strains by comparison with the *P. aeruginosa* PAO1.

Gene	Gene Description	Amino Acid Change ^a^					
		R1	R2	R3	C1	C2	C3
*gyrA*	DNA gyrase subunit A	T83I	T83A	T83V	-	-	-
*gyrB*	DNA gyrase subunit B	-	A458AP	-	-	-	-
*parC*	topoisomerase IV subunit A	E91K	-	S87L	-	-	-
*parE*	topoisomerase IV subunit B	D450EVD	G451VDG	R586W	-	-	-
*nfxB*	transcriptional regulator *NfxB*	X188C	X188C	G180S	-	-	-
PA0625	hypothetical protein	-	-	L659fs	-	-	-

^a^ same as that in PAO1 (www.pseudomonas.com, accessed on 6 January 2021).

## Data Availability

The raw sequence data of genomic DNA reference mapping have been deposited in the NCBI with accession number, PRJNA875020 (www.ncbi.nlm.nih.gov/sra/?term=PRJNA875020+, accessed on 30 August 2022).

## Supplementary Materials

The following supporting information can be downloaded at: https://www.mdpi.com/article/10.3390/microorganisms11010013/s1. Figure S1: Development of resistance against ciprofloxacin by clinical isolate CRP42. MICs of experimentally evolved mutants to ciprofloxacin. Three lineages were passaged in the presence of ciprofloxacin for experimental evolution. The MICs of ciprofloxacin were measured daily. Table S1: Bacterial strains and plasmids used in this study. Table S2: Primers used in this study. Table S3: Gene mutations identified by genome reference mapping with elimination of SNVs with heterozygous mutations, synonymous mutations, and the same mutations between evolved strains and control strains. Reference [28] is cited in the supplementary materials.

## References

[1] de Bentzmann S., Plésiat P. (2011). The *Pseudomonas aeruginosa* opportunistic pathogen and human infections. Environ. Microbiol..

[2] Tacconelli E., Carrara E., Savoldi A., Harbarth S., Mendelson M., Monnet D.L., Pulcini C., Kahlmeter G., Kluytmans J., Carmeli Y. (2018). Discovery, research, and development of new antibiotics: The WHO priority list of antibiotic-resistant bacteria and tuberculosis. Lancet Infect. Dis..

[3] Boucher H.W., Talbot G.H., Bradley J.S., Edwards J.E., Gilbert D., Rice L.B., Scheld M., Spellberg B., Bartlett J. (2009). Bad bugs, no drugs: No ESKAPE! An update from the Infectious Diseases Society of America. Clin. Infect. Dis. Off. Publ. Infect. Dis. Soc. Am..

[4] Rehman A., Patrick W.M., Lamont I.L. (2019). Mechanisms of ciprofloxacin resistance in *Pseudomonas aeruginosa*: New approaches to an old problem. J. Med. Microbiol..

[5] Pitt T.L., Sparrow M., Warner M., Stefanidou M. (2003). Survey of resistance of *Pseudomonas aeruginosa* from UK patients with cystic fibrosis to six commonly prescribed antimicrobial agents. Thorax.

[6] Lee J.K., Lee Y.S., Park Y.K., Kim B.S. (2005). Alterations in the *GyrA* and *GyrB* subunits of topoisomerase II and the *ParC* and *ParE* subunits of topoisomerase IV in ciprofloxacin-resistant clinical isolates of *Pseudomonas aeruginosa*. Int. J. Antimicrob. Agents.

[7] Pasca M.R., Dalla Valle C., De Jesus Lopes Ribeiro A.L., Buroni S., Papaleo M.C., Bazzini S., Udine C., Incandela M.L., Daffara S., Fani R. (2012). Evaluation of fluoroquinolone resistance mechanisms in *Pseudomonas aeruginosa* multidrug resistance clinical isolates. Microb. Drug Resist..

[8] Xu C., Liu H., Pan X., Ma Z., Wang D., Zhang X., Zhu G., Bai F., Cheng Z., Wu W. (2020). Mechanisms for Development of Ciprofloxacin Resistance in a Clinical Isolate of *Pseudomonas aeruginosa*. Front. Microbiol..

[9] Chávez-Jacobo V.M., Hernández-Ramírez K.C., Romo-Rodríguez P., Pérez-Gallardo R.V., Campos-García J., Gutiérrez-Corona J.F., García-Merinos J.P., Meza-Carmen V., Silva-Sánchez J., Ramírez-Díaz M.I. (2018). CrpP Is a Novel Ciprofloxacin-Modifying Enzyme Encoded by the *Pseudomonas aeruginosa* pUM505 Plasmid. Antimicrob. Agents Chemother..

[10] Cabot G., Zamorano L., Moyà B., Juan C., Navas A., Blázquez J., Oliver A. (2016). Evolution of *Pseudomonas aeruginosa* Antimicrobial Resistance and Fitness under Low and High Mutation Rates. Antimicrob. Agents Chemother..

[11] Wong A., Rodrigue N., Kassen R. (2012). Genomics of adaptation during experimental evolution of the opportunistic pathogen *Pseudomonas aeruginosa*. PLoS Genet..

[12] Clinical and Laboratory Standards Institute (2009). Methods for Dilution Antimicrobial Susceptibility Tests for Bacteria That Grow Aerobically; Approved Standard.

[13] Hoang T.T., Karkhoff-Schweizer R.R., Kutchma A.J., Schweizer H.P. (1998). A broad-host-range Flp-FRT recombination system for site-specific excision of chromosomally-located DNA sequences: Application for isolation of unmarked *Pseudomonas aeruginosa* mutants. Gene.

[14] Ma Z., Xu C., Zhang X., Wang D., Pan X., Liu H., Zhu G., Bai F., Cheng Z., Wu W. (2021). A MexR Mutation Which Confers Aztreonam Resistance to *Pseudomonas aeruginosa*. Front. Microbiol..

[15] Bruchmann S., Dötsch A., Nouri B., Chaberny I.F., Häussler S. (2013). Quantitative contributions of target alteration and decreased drug accumulation to *Pseudomonas aeruginosa* fluoroquinolone resistance. Antimicrob. Agents Chemother..

[16] Wardell S.J.T., Rehman A., Martin L.W., Winstanley C., Patrick W.M., Lamont I.L. (2019). A large-scale whole-genome comparison shows that experimental evolution in response to antibiotics predicts changes in naturally evolved clinical *Pseudomonas aeruginosa*. Antimicrob. Agents Chemother..

[17] Purssell A., Poole K. (2013). Functional characterization of the *NfxB* repressor of the mexCD-oprJ multidrug efflux operon of *Pseudomonas aeruginosa*. Microbiology.

[18] Monti M.R., Morero N.R., Miguel V., Argaraña C.E. (2013). *nfxB* as a novel target for analysis of mutation spectra in *Pseudomonas aeruginosa*. PLoS ONE.

[19] Michel-Briand Y., Baysse C. (2002). The pyocins of *Pseudomonas aeruginosa*. Biochimie.

[20] Nakayama K., Takashima K., Ishihara H., Shinomiya T., Kageyama M., Kanaya S., Ohnishi M., Murata T., Mori H., Hayashi T. (2000). The R-type pyocin of *Pseudomonas aeruginosa* is related to P2 phage, and the F-type is related to lambda phage. Mol. Microbiol..

[21] Akasaka T., Tanaka M., Yamaguchi A., Sato K. (2001). Type II topoisomerase mutations in fluoroquinolone-resistant clinical strains of *Pseudomonas aeruginosa* isolated in 1998 and 1999: Role of target enzyme in mechanism of fluoroquinolone resistance. Antimicrob. Agents Chemother..

[22] Brazas M.D., Hancock R.E. (2005). Ciprofloxacin induction of a susceptibility determinant in *Pseudomonas aeruginosa*. Antimicrob. Agents Chemother..

[23] Breidenstein E.B., Khaira B.K., Wiegand I., Overhage J., Hancock R.E. (2008). Complex ciprofloxacin resistome revealed by screening a *Pseudomonas aeruginosa* mutant library for altered susceptibility. Antimicrob. Agents Chemother..

[24] Levin-Reisman I., Ronin I., Gefen O., Braniss I., Shoresh N., Balaban N.Q. (2017). Antibiotic tolerance facilitates the evolution of resistance. Science.

[25] Chen F., Chen G., Liu Y., Jin Y., Cheng Z., Liu Y., Yang L., Jin S., Wu W. (2017). *Pseudomonas aeruginosa* Oligoribonuclease Contributes to Tolerance to Ciprofloxacin by Regulating Pyocin Biosynthesis. Antimicrob. Agents Chemother..

[26] Long Y., Fu W., Wang S., Deng X., Jin Y., Bai F., Cheng Z., Wu W. (2020). Fis Contributes to Resistance of *Pseudomonas aeruginosa* to Ciprofloxacin by Regulating Pyocin Synthesis. J. Bacteriol..

[27] Fan Z., Chen H., Li M., Pan X., Fu W., Ren H., Chen R., Bai F., Jin Y., Cheng Z. (2019). *Pseudomonas aeruginosa* Polynucleotide Phosphorylase Contributes to Ciprofloxacin Resistance by Regulating PrtR. Front. Microbiol..

[28] Kaufman M.R., Jia J., Zeng L., Ha U., Chow M., Jin S. (2000). *Pseudomonas aeruginosa* mediated apoptosis requires the ADP-ribosylating activity of exoS. Microbiology.

