# Supplementary material for: Parallel Evolution to Elucidate the Contributions of PA0625 and parE to Ciprofloxacin Sensitivity in Pseudomonas aeruginosa
Source: Microorganisms. 2022 Dec 21;11(1):13. doi: 10.3390/microorganisms11010013 (PMC9860795; doi:10.3390/microorganisms11010013)
Supplement: Supplementary file 1 [file microorganisms-11-00013-s001.zip › Table S1_strains.docx]

**Table S1.** **Bacterial strains and plasmids used in this study.**

| Strain or plasmid | | Description | Source or reference |
| --- | --- | --- | --- |
| **strains** | |  |  |
| ***E. coli* strains** | |  |  |
| DH5α | | F^̶^ ϕ 80d*lacZ*∆M15 *endA1 recA1 hsdR17*(r_K_^̶^ m_K_^+^) *supE44 thi-1 relA1* ∆(*lacZYA-argF*) *U169 gyrA96 deoR* | TransGen |
| S17-1 | RP4-2 Tc::Mu Km::Tn*7* Tp^r^ Sm^r^ Pro Res^̶^ Mod^+^ | | Dr. Ramphal |
| ***P. aeruginosa* strains** |  | |  |
| PAO1 | Wild type *P. aeruginosa* strain | | 28 |
| CRP42 | A ciprofloxacin resistant clinical isolate | | 8 |
| CSP18*mexS*_CRP42_ | CSP18 with its *mexS* replaced by that of CRP42 | | 8 |
| R1 | Resistant strain 1 of parallel evolution in the presence of ciprofloxacin | | This study |
| R2 | Resistant strain 2 of parallel evolution in the presence of ciprofloxacin | | This study |
| R3 | Resistant strain 3 of parallel evolution in the presence of ciprofloxacin | | This study |
| C1 | Control strain 1 of parallel evolution in the absence of antibiotic | | This study |
| C2 | Control strain 2 of parallel evolution in the absence of antibiotic | | This study |
| C3 | Control strain 3 of parallel evolution in the absence of antibiotic | | This study |
| CRP42*nfxB*^G180S^ | The 180^th^ amino acid of NfxB changed from G to S in CRP42 | | This study |
| CRP42*nfxB*^X188C^ | The stop codon of NfxB changed to C in CRP42 | | This study |
| CSP18*mexS*_CRP42_*nfxB*^G180S^ | The 180^th^ amino acid of NfxB changed from G to S in CSP18*mexS*_CRP42_ | | This study |
| CSP18*mexS*_CRP42_*nfxB*^X188C^ | The stop codon of NfxB changed to C in CSP18*mexS*_CRP42_ | | This study |
| CRP42*parE*^R586W^ | The 586^th^ amino acid of ParE changed from R to W in CRP42 | | This study |
| PAO1*parE*^R586W^ | The 586^th^ amino acid of ParE changed from R to W in PAO1 | | This study |
| CRP42PA0625_R3_ | Simulate the mutation of PA0625 of R3 in CRP42 | | This study |
| CRP42ΔPA0625 | PA0625 deleted in CRP42 | | This study |
| PAO1PA0625_R3_ | Simulate the mutation of PA0625 of R3 in PAO1 | | This study |
| PAO1ΔPA0625 | PA0625 deleted in PAO1 | | This study |
| **Plasmids** |  | |  |
| pEX18Tc | Gene replacement vector; Tc^r^, *oriT*^+^, *sacB*^+^ | | 13 |
| pEX18-*nfxB*^G180S^ | pEX18Tc with the *nfxB* gene of R3; Tc^r^ | | This study |
| pEX18-*nfxB*^X188C^ | pEX18Tc with the *nfxB* gene of R1; Tc^r^ | | This study |
| pEX18- *parE*^R586W^ | pEX18Tc with the *parE* gene of R3; Tc^r^ | | This study |
| pEX18- PA0625_R3_ | pEX18Tc with the PA0625 gene of R3; Tc^r^ | | This study |
| pEX18-PA0625-1 | pEX18Tc with the PA0625 gene of PAO1; Tc^r^ | | This study |
| pEX18-PA0625-2 | pEX18Tc with the PA0625 gene of CRP42; Tc^r^ | | This study |
