# Supplementary material for: Parallel Evolution to Elucidate the Contributions of PA0625 and parE to Ciprofloxacin Sensitivity in Pseudomonas aeruginosa
Source: Microorganisms. 2022 Dec 21;11(1):13. doi: 10.3390/microorganisms11010013 (PMC9860795; doi:10.3390/microorganisms11010013)
Supplement: Supplementary file 1 [file microorganisms-11-00013-s001.zip › Table S2_primer.docx]

**Table S2. Primers used in this study.**

| Primer^a^ | Sequence 5’-3’ | Use | Reference/Source |
| --- | --- | --- | --- |
| *gyrA*-F | CGACCTACTTCGTTTGCCTCAG | Gene amplification for verification of reference mapping | This study |
| *gyrA*-R | GCTCGCTTGCTCACATCCAC |  |  |
| *gyrB*-F | GACCCGCAACTATTGAAAGAC |  |  |
| *gyrB*-R | CAACGATCGACCGAACCGAC |  |  |
| *parC*-F | GGCGATGAGGTGCTACTCGG |  |  |
| *parC*-R | CCGCCAGGGAGACGACATT |  |  |
| *parE*-F | GCAGACCGCCGACGAAA |  |  |
| *parE*-R | ATGCCCTCCACCGCATG |  |  |
| *nfxB*-F | ACGCGAGGCCAGTTTTCT |  |  |
| *nfxB*-R | ACTGATCTTCCCGAGTGTCG |  |  |
| *parE*-FF | CGAGCTCGATGCAGGAGCCGCAGTT | *parE* for gene editing and test | This study |
| *parE*-RR | CCCAAGCTTCAGCGCCAGGCACTGTT |  |  |
| t-*parE* | GTCGATCCGGGTGCCAGTGAC |  |  |
| *nfxB-*FF | CGGGGTACCGAAGCGCTTCCGCACGACG GCTG | *nfxB* for gene editing and test | This study |
| *nfxB*-RR | CGCGGATCCCGCTGTTGCGCAGCGGT |  |  |
| t-*nfxB* | GTGCAGATGCTCGAGGACCAC |  |  |
| PA0625-F | GGAATTCCGCAGACCGCTTTGGGCAG | PA0625 for verification, gene editing, and test | This study |
| PA0625-R | CCCAAGCTTTCAGGTTGAAGTAGAACGGTTG |  |  |
| t-PA0625 | AGTGGCCTCGTCGTTGATC |  |  |

a: F, forward; R, reverse; t, test.
