# Supplementary figures and images for: Parallel Evolution to Elucidate the Contributions of PA0625 and parE to Ciprofloxacin Sensitivity in Pseudomonas aeruginosa
Source: Microorganisms. 2022 Dec 21;11(1):13. doi: 10.3390/microorganisms11010013 (PMC9860795; doi:10.3390/microorganisms11010013)

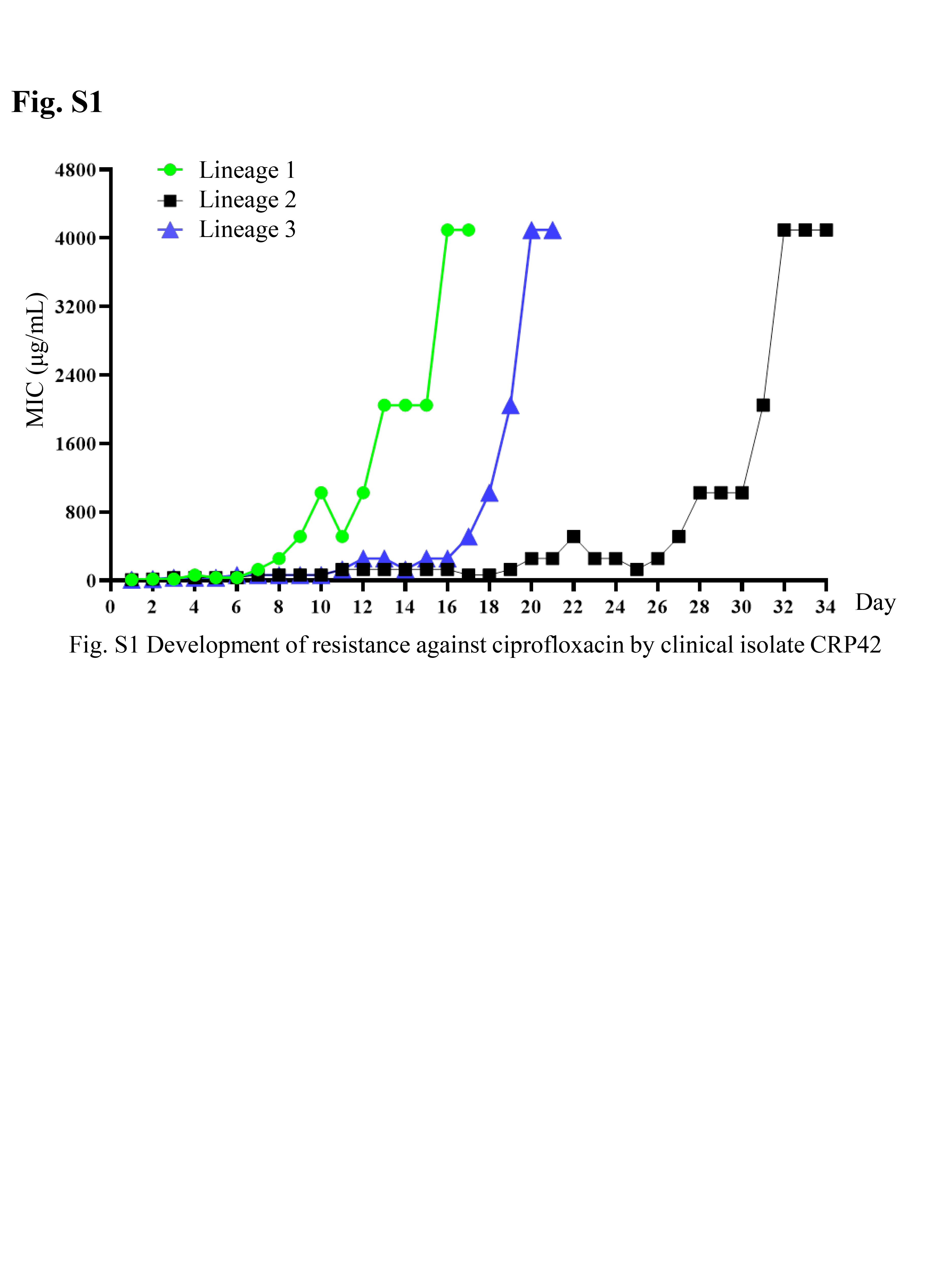

Supplement: Supplementary file 1 [file microorganisms-11-00013-s001.zip › Fig S1.tif]
